# Enrofloxacin Promotes Plasmid-Mediated Conjugation Transfer of Fluoroquinolone-Resistance Gene *qnrS*

**DOI:** 10.3389/fmicb.2021.773664

**Published:** 2022-02-16

**Authors:** Yue Zhao, Zhengzheng Cao, Luqing Cui, Tianyu Hu, Kaixuan Guo, Fan Zhang, Xiangru Wang, Zhong Peng, Quan Liu, Menghong Dai

**Affiliations:** ^1^The Co-operative Innovation Center for Sustainable Pig Production, Huazhong Agricultural University, Wuhan, China; ^2^Ministry of Agriculture (MOA) Key Laboratory of Food Safety Evaluation/National Reference Laboratory of Veterinary Drug Residue (HZAU), Huazhong Agricultural University, Wuhan, China

**Keywords:** *qnrS*, *Escherichia coli E2*, *Salmonella* enterica serovar enteritidis SE211, enrofloxacin, resistance transfer

## Abstract

This study aimed to determine the effect of enrofloxacin (ENR) on the transfer of the plasmid-mediated quinolone resistance (PMQR) gene *qnrS* from opportunistic pathogen *Escherichia coli* (E2) to *Salmonella* Enteritidis (SE211) and to analyze the resistance characteristics of SE211-*qnrS* isolates. The plasmid carrying *qnrS* gene of E2 was sequenced by Oxford Nanopore technology. The plasmid carrying *qnrS* gene belonged to incompatibility group IncY. *In vitro*, the transfer experiment of IncY plasmid was performed by the liquid medium conjugation method. The conjugation transfer frequency of the IncY plasmid was 0.008 ± 0.0006 in the absence of ENR, 0.012 ± 0.003 in 1/32 MIC_ENR_, 0.01 ± 0.008 in 1/8 MIC_ENR_, and 0.03 ± 0.015 (Mean±SD) in 1/2 MIC_ENR_, respectively. After inoculation of *E. coli* E2 and SE211, chickens were treated with different doses of ENR (3.03, 10, and 50 mg/kg b.w.) for 7 days consecutively. To screen the SE211-*qnrS* strains from intestinal tract of chickens, the resistance genes and susceptibility of isolates were identified. The amount of *E. coli* E2 and the copy number of *qnrS* gene in the chicken intestinal tract were determined by colony counting and qPCR, respectively. *In vivo*, more SE211-*qnrS* strains were isolated from the treated group compared with the untreated group. SE211-*qnrS* strains not only obtained IncY plasmid, but also showed similar resistance phenotype as E2. In conclusion, ENR treatment can promote the spread of a IncY-resistance plasmid carrying the *qnrS* fluoroquinolone-resistance gene in *Escherichia coli* and the development of drug-resistant bacteria.

## Introduction

*Escherichia coli* (abbreviated as *E. coli*) is a common opportunistic pathogen in the gastrointestinal tract. An estimated 44 million ETEC-related diarrheal diseases occur annually, resulting in 113,000 deaths in 2015 (Roussel et al., 2020), and the mortality is 3–5% in *E. coli* infection.^1^ Non-typhoidal *Salmonellae*, one of the leading causes of bacterial diarrhea worldwide, are estimated to cause approximately 153 million cases of gastroenteritis and 57,000 deaths globally each year.^2^ Fluoroquinolones (FQs) are commonly used in the treatment of colibacillosis and salmonellosis and can induce resistance of intestinal bacteria (Li J. et al., 2019). *QnrS* gene, a plasmid-mediated FQ-resistance gene, was the most prevalent quinolone gene in *E. coli* strains isolated from poultry feces (Gosling et al., 2012). Antibiotic resistance genes (ARGs) can be the agent of an outbreak by transferring resistance to multiple unrelated pathogens (Lerminiaux and Cameron, 2019). In the case of antibiotic abuse, whether opportunistic pathogens transfer ARGs to pathogens has attracted widespread attention. *E. coli* was used as a vector to transmit the *qnrS* gene, which could make pathogenic bacteria obtain drug-resistance genes and reach the level of clinical drug resistance (Gosling et al., 2012). Furthermore, the plasmid carrying *qnrS* gene in *E. coli* also contained other ARGs (Veldman et al., 2014; Slettemeas et al., 2019; Koyama et al., 2020), which could cotransfer with the *qnrS* gene. *Salmonella* Enteritidis 211 (abbreviated as SE211) is highly pathogenic (Cui et al., 2020, 2021), and it is sensitive to enrofloxacin. *E. coli* E2 carrying *qnrS* gene is a multidrug resistant (MDR) strain (Li et al., 2017). Previous research results show that the *qnrS* gene promotes the transfer efficiency of ARGs from *E. coli* E2 to *E. coli* strain EC600 *in vitro*, suggesting the intraspecies transfer capacity of resistance genes (opportunistic pathogen *E. coli* E2 to model recipient *E. coli* EC600). However, the interspecies transfer (opportunistic pathogen *E. coli* to pathogenic *Salmonella*) capacity of the *qnrS*-carrying *E. coli* under the antibiotic selection pressure in broiler chicken was unexplored. ENR can favor the broiler gastrointestinal (GI) tract acting as a niche for selection of MDR commensal coliforms (Li et al., 2017). Furthermore, the intestinal tract of chicken provides a habitat for different bacteria. The multifactorial and complex relationships of the microbiota ecosystem of the gut contribute to the spread of these bacteria between animals and humans (Tewari et al., 2019). Therefore, we analyzed the effect of enrofloxacin on the transfer of *qnrS* gene from *E. coli* E2 to SE211 both *in vitro* and *in vivo* and the resistance characteristic of SE211 carrying *qnrS* gene (SE211-*qnrS*). The transfer of *qnrS* gene from *E. coli* E2 to SE211 in the absence of ENR and in the presence of subminimal inhibitory concentration (sub-MIC) of ENR was performed by the liquid medium conjugation method. The chickens were gavaged with a bacteria suspension of *E. coli* E2 and SE211 before ENR administration. SE211-*qnrS* isolates were screened on plates supplemented with ENR. The colonization levels of *E. coli* E2 and SE211-*qnrS*, copy number of *qnrS*, were determined by traditional culture methods and molecular biology identification techniques.

## Materials and Methods

### Ethics

The study *in vivo* was carried out in accordance with the guidelines established by the China Regulations for the Administration of Affairs Concerning Experimental Animals (1988) and Regulations for the Administration of Affairs Concerning Experimental Animals in Hubei province (2005) (Project No.2017YFC1600100 and Animal Welfare Assurance No. HZAUCH-2020-0005). All work was made to treat the experimental animals ethically and to minimize suffering in this study.

### Experimental Strains and Reagents

#### Donor and Recipient Bacteria

*E. coli* E2 containing the *qnrS* gene was used as a donor strain (MIC_ENR_ = 128 mg/L). *Salmonella* SE211 was used as a recipient strain (MIC_ENR_ = 0.25 mg/L). They were preserved by the Cooperative Innovation Center for Sustainable Pig Production (HZAU), Huazhong Agricultural University.

#### Reagents

The standards of enrofloxacin, chloramphenicol, ampicillin, trimethoprim, and sulfamethoxazole were purchased from Dr. Ehrenstorfer (Germany). Ciprofloxacin standard was obtained from MedChemExpress (New Jersey, United States). Tetracycline standard was from China Institute of Veterinary Drug Control (Beijing, China), and 2 × EasyTaq PCR SuperMix and Phanta super-fidelity DNA Polymerase were purchased from Vazyme (Nanjing, China). The pUCm-T vector, IPTG, and X-Gal were from Beyotime Biotechnology (Nantong, China). The primers of this study were all synthesized by Genscript (Nanjing, China).

### Plasmid Sequencing and Analysis

*E. coli* E2 was grown in LB broth, and the culture was centrifuged and quickly frozen in liquid nitrogen. Relying on Wuhan Bena Technology Service Limited Company, the genomic DNA was extracted by sodium dodecyl sulfate (SDS) and purified with a 13323 kit. The plasmid sequencing was performed by the Oxford Nanopore Technologies DNA sequencing platform. Annotation of plasmid type and resistance genes were carried out on the website.^3^ According to the plasmid sequencing result, NCBI was used to design the primers for plasmid replicon and resistance genes. Comparison of IncY plasmids was created by BRIG tools.^4^ The comparison of IncY plasmids was performed in the following order (inner to outer circles): pTET-GZEC065 (GenBank accession no. CP048027), pTetA_020022 (CP032890), pE2 (CP086663).

### Conjugation Transfer and Construction of Animal Model

#### Transfer of *qnrS in vitro*

The conjugation experiment was conducted using the liquid mating procedure according to previous report (Lambrecht et al., 2018). *E. coli* E2 and SE211 with 4:1 volume ratio were cocultivated for 4 h in LB broth supplemented with 0, 1/2 MIC_ENR_ (0.0125 mg/L), 1/8 MIC_ENR_ (0.003 mg/L), and 1/32 MIC_ENR_ (0.0007 mg/L). The coculture was diluted 10^4^ times by LB broth and spread on the chromogenic *Salmonella* agar (second generation) (Hopebio, Qingdao, China). The plates were supplemented with 0.25 mg/L of ENR to screen the SE211-*qnrS* strains. The conjugation transfer efficiency = $\frac{Numberoftransconjugants}{Numberofrecipients}$.

#### Chickens and Housing

The animal trial was performed in the animal room at Ke Qian of HuaZhong Agricultural University. Twenty specific-pathogen-free (SPF) male chickens (1-day-old) were purchased from Bejing Boehringer Ingelheim Vital Biotechnology Limited Company and kept in four individual PQ3 type stainless steel poultry isolators (Suzhou Suhang Technology Equipment Co., Ltd., China), which can prevent pollution from external environmental factors. They were fed with sterile water and SPF feed (Beijing Ke Ao Xie Li Feed Limited Company, China).

#### Model Construction and Enrofloxacin Treatment

**FIGURE 1:**
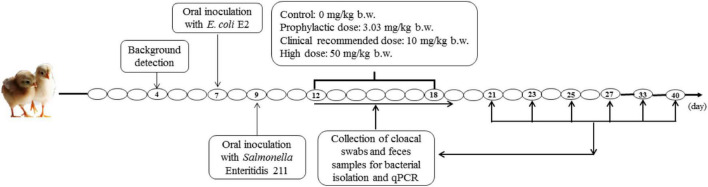
Experimental design. The 12–18 days represent ENR treatment for 7 days. The 21, 23, 25, 27, 33, 40 days represent 2, 4, 6, 8, 14, 21 days after termination of ENR treatment, respectively.

On day 7, all chickens were inoculated with 0.5 mL of the donor strain (∼10^9^ CFU/mL). On day 10, all chickens were inoculated with 0.5 mL of the recipient strain (∼10^9^ CFU/mL). The chickens were gavaged with bacterial suspension. After successful colonization of the intestine with the donor and recipient bacteria on day 12, the 20 chickens were equally divided into four groups. Then, they were treated with different dosages of ENR (10% enrofloxacin oral solution, Bayer, Germany). The first group (group 3.03) was given a prophylactic dose (3.03 mg/kg b.w.) (Li et al., 2017). The second group (group 10) was given the ENR (10 mg/kg b.w.) as a clinical recommended dose. The third group (group 50) was given a high dose (50 mg/kg b.w.), which could effectively inhibit pathogenic bacteria. The fourth group did not receive treatment and served as a non-treated control group (group NTC). The different dosages of ENR were given for 7 days consecutively (Figure 1).

### Sample Collection and Strain Isolation

#### Collection of Fecal and Cloacal Samples

Prior to the inoculation with *E. coli* E2 and SE211, cloacal swabs were taken from each chicken, then cultivated on chromogenic *Salmonella* agar (second generation) plates and in LB broth (Hopebio, Qingdao, China). *QnrS* and *repA* genes were identified by PCR. None of the chickens was found to be positive for *Salmonella*, resistance gene *qnrS*, and IncY plasmid. No contamination of *Salmonella*, IncY plasmid, and *qnrS* gene were observed in the drinking water and SPF feed. Cloacal swabs and fecal samples were collected on day 12 (1 day before ENR treatment); days 13–19 (2, 4, 6, 8 days of the treatment of ENR); and days 21, 23, 25, 27, 33, and 40 (1 and 2 weeks after stopping ENR treatment), respectively (Figure 1).

#### Bacterial Isolation

Two swabs were taken from each chicken. One swab was emulsified in 2 mL of selenite cystine broth (SC) (Hopebio, Qingdao, China) and then further inoculated on chromogenic *Salmonella* agar (second generation) plates supplemented with 0.25 mg/L of ENR. *Salmonella* represented a typical purple single colony on the chromogenic *Salmonella* agar (second generation). The colonies were randomly screened for further amplification of *qnrS* and *repA* genes to identify SE211-*qnrS* strains. In the meantime, all the *Salmonella* and putative SE211-*qnrS* isolates were stored at –20°C. Another swab was weighed, which was emulsified in 1 mL of sterile 0.9% NaCl and then further diluted 10^5^ times. An aliquot (50 μL) of the appropriate dilution was spread onto eosin-methylene blue agar (Hopebio, Qingdao, China) plates supplemented with ENR (32 mg/L). These plates were incubated at 37°C overnight to detect the colonization level of *E. coli* E2.

### Quantitation PCR

#### Standard Curve

Snapgene software (Version 3.1.1) was used to design primers (5′–3′) for amplification of the full length (657 bp) of *qnrS* gene (F-5′-ATGGAAACCTACAATCATACATATCGG-3′ and R-5′-TTAGTCAGGATAAACAACAATACCC-3′). PCR product of *qnrS* gene was obtained by recycling from 1% agarose gels. Then, the recombinant DNA of pUCm-T-*qnrS* was obtained by ligation and transformation (DH5α). The plasmid pUCm-T-*qnrS* was extracted with E.Z.N.A.^®^ Plasmid Mini Kit II (Wuhan Tianyuan Huida Biotechnology Limited Company, China). The copy number of *qnrS* gene was calculated by the equation: Copy number (copies/μL) = 6.02 × 10^23^ × cDNA (g/mL)/MW, molecular weight (MW) = (plasmid vector length + insertion fragment length) × 660 dalton/bp. Then, pUCm-T-*qnrS* of 1.17 × 10^8^, 1.17 × 10^7^, 1.17 × 10^6^, 1.17 × 10^5^, and 1.17 × 10^4^ copies were used as the standards to determine the corresponding CT values. CT = –KlgC_0_+b was used as the standard curve formula, and C_0_ represented the copy number of *qnrS* gene.

#### DNA Isolation

Total DNA was extracted from 210 mg of fecal samples collected from chickens using by commercial extraction kits (Rapid extraction kit for fecal genomic DNA, Aidlab, Beijing, China) according to the manufacturer’s instruction and our modification. We increased the number of samples through the AC adsorption column and the proteinase K.

#### Analysis of the Quantities of the *qnrS* Gene

Quantitative PCR (qPCR) was performed by SYBR Green (Vazyme, Nanjing, China) detection in triplicate using a QuantStudio 3 real-time PCR detection system (Thermo Fisher Scientific, Germany) by the following procedure: 1 cycle at 95°C for 30 s, 40 cycles at 95°C for 10 s, 57°C for 10 s, 72°C for 30 s. The specificity of the PCR products was confirmed by melting curve. The copy number was calculated by equation of standard curve.

#### Verification of SE211-*qnrS*

All of the isolates were confirmed to be SE211 strains and then tested the IncY plasmid by the PCR method using the primer pairs (5′–3′) for *qnrS* (F-5′-CATACATATCGGCACCACAAC-3′ and R-5′-CAGGATAAACAACAATACCCAGT-3′), *repA* (F-5′-AATTCAAACAACACTGTGCAGCCTG-3′ and R-5′-GCGAGAATGGACGATTACAAAACTTT-3′). All of the SE211-*qnrS* strains were tested for the resistance genes located in the IncY plasmid by PCR method using the primers (5′–3′) for *bla*_TEM–135_ (F-5′-TTGATCGTTGGGAACCGGAG-3′ R-5′-AAT AAACCAGCCAGCCGGAA-3′), *tet*(A) (F-5′-CATTTCGCTT GCCGCATTTG-3′ and R-5′-TCATTCCGAGCATGAGTGCC-3′), *floR* (F-5′-CGGATTCAGCTTTGCCTTCG-3′ R-5′-GCCAA TGTCCCGACGATACT-3′), and *dfrA*-14 (F-5′-CAACGATG TTACGCAGCAGG-3′ R-5′-CAATCGCGGAAAAGGCG TAG-3′).

### Susceptibility Tests

MICs of four antibiotics (enrofloxacin, ampicillin, chloramphenicol, tetracycline) for all SE211-*qnrS* strains were determined by the broth microdilution method, and their antibiotic resistance level was interpreted by the Clinical and Laboratory Standards Institute guidelines (CLSI) (CLSI, 2020). In particular, the resistant breakpoint of ampicillin, chloramphenicol, and tetracycline for *Salmonella* strains was interpreted by the CLSI criteria (ampicillin ≥ 32 mg/L, chloramphenicol ≥ 32 mg/L, tetracycline ≥ 16 mg/L), whereas no resistant breakpoint of ENR was interpreted. Ciprofloxacin was interpreted based on the CLSI breakpoint (R ≥ 1 mg/L). *E. coli* ATCC 25,922 served as a quality control strain.

### Stability of Plasmid

In this study, the plasmid stability experiment was different from the other report (Wein et al., 2019), which was evaluated by serial passages. In this study, we aimed to obtain colonies of SE211-*qnrS* from the glycerol bacteria. Method 1: The SE211-*qnrS* glycerol bacteria were cultured in LB broth supplemented with ENR (0.25 mg/L) at 37°C for 12–16 h, and then 20 μL culture was spread on chromogenic *Salmonella* agar (second generation) plates supplemented with ENR (1 mg/L) and incubated at 37°C for 18–24 h. Then, colonies were randomly selected on the resistant plates as a template for PCR identification. Method 2: The glycerol bacteria of SE211-*qnrS* was supplemented with ENR (0.25 mg/L) and stored in –20°C for 48 h. Cells of SE211-*qnrS* from the glycerol stock were supplemented with ENR (0.25 mg/L) and stored at –20°C for 48 h. The cells were cultured in LB broth supplemented with ENR (0.25 mg/L) at 37°C for 12 h, and 50 μL of the culture was spread on LB agar plates supplemented with (0.25 mg/L) and incubated again at 37°C for 12 h. Cells were harvested from these plates and resuspended in LB broth supplemented with (0.25 mg/L) and used as a template for PCR. *QnrS* and *repA* genes were amplified from resuscitative and eluotropic bacteria solutions to identify the SE211-*qnrS* strains.

### Statistical Analysis

Using Graphpad Prism 7.0 statistical software, the value was expressed by mean ± SD, and the differences among different time points were analyzed by Student’s *t*-test. *P ≤ 0.05 was set as the significance level, and ^**^P ≤ 0.01 was set as the extremely significance difference.

## Results

### Genetic Characteristics of IncY Plasmids

**FIGURE 2:**
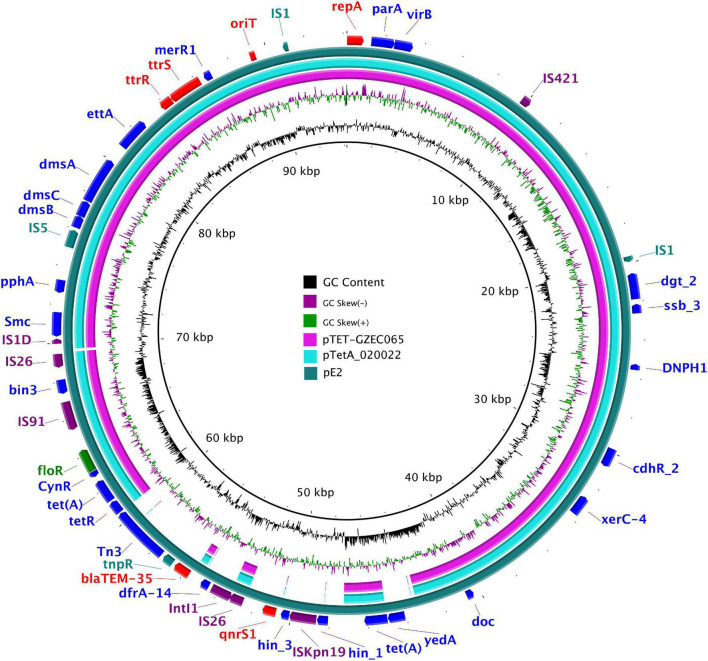
The complete sequence of pE2 (the outer circle) was used as a reference plasmid. The circular maps were generated using the BRIG software, and plasmids were included in the following order (inner to outer circles): pTET-GZEC065 (CP048027), pTetA_020022 (CP032890.1), pE2 (CP086663).

The plasmid (pE2) harbored in *E. coli* E2 had a size of 94, 190 bp with an average G + C content of 49.59%. The replicon gene *repA* showed 100% identity with the replicon of IncY plasmids in the PlasmidFinder database. The plasmid distribution system *parA* and *virB* genes were associated with the self-transmitting of IncY plasmids. An *oriT* sequence was found between the positions 89,095 and 89,379 by *oriTfinder*. Multiple mobile elements were distributed on pE2, such as insertion sequences (IS) IS421, IS1, ISKpn19, IS26, IS5, IS91; integron functional element IntI1; and transposon Tn3. Additional resistance genes were found on the plasmid, such as *tet*(A), *dfrA-*14, *bla*_TEM–135_, and *floR.* The sequence of plasmid pE2 was highly similar to that of pTET-GZEC065 and pTetA_020022 (85% query coverage and 100% sequence identity). These plasmids all contained the *floR* and *tet*(A) resistance genes, including insertion elements (Figure 2).

### The Effect of Subminimal Inhibitory Concentration of Enrofloxacin on the Transfer Frequency of *qnrS* Gene *in vitro*

No significant changes in the transfer frequency of *qnrS* gene from resistant *E. coli* E2 to susceptible SE211 was observed under 0, 1/2 MIC_ENR_ (0.125 mg/L), 1/8 MIC_ENR_ (0.03 mg/L), and 1/32 MIC_ENR_ (0.007 mg/L) conditions. However, the transfer frequency increased with increasing concentration of ENR (Table 1).

**TABLE 1:** Transfer frequency of *qnrS* gene under sub-MIC of ENR.

Concentration of ENR	Transfer frequency
0	0.008 ± 0.0006
1/32 MIC_ENR_	0.012 ± 0.003
1/8 MIC_ENR_	0.01 ± 0.008
1/2 MIC_ENR_	0.03 ± 0.015

*Three replicates in each group, the transfer frequency was analyzed by Student’s t-test.*

### The Effect of Enrofloxacin on the Persistence and the Transfer of *qnrS* Gene *in vivo*

**FIGURE 3:**
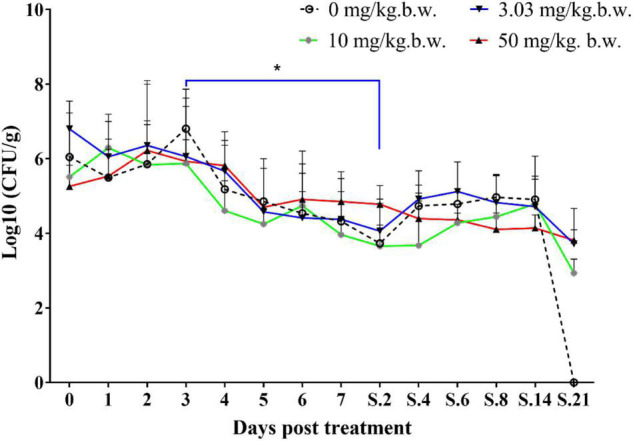
Colonization level of drug-resistant *E. coli* E2 in chicken intestine. The 0–7 represent days for ENR treatment. S represents days after termination of ENR treatment. S.2 represents 2 days after termination of ENR treatment. The *p ≤ 0.05 that was set as significance level.

**FIGURE 4:**
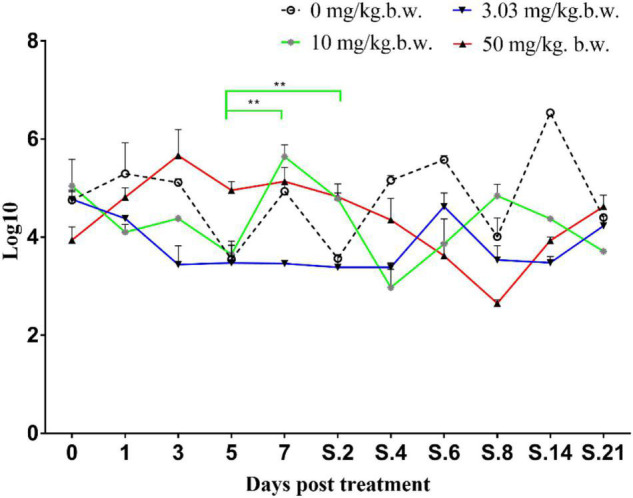
Copy number of *qnrS* gene in fecal genome. The 0–7 represent days for ENR treatment. S represents days after termination of ENR treatment. S.2 represents 2 days after termination of ENR treatment. The **p ≤ 0.01 that was set as extremely significance difference.

Prior to inoculation, cloacal swabs of the chickens showed no *E. coli* carrying *qnrS* gene and *Salmonella* as judged by the EMB agar plates supplemented with ENR (32 mg/L) and the chromogenic *Salmonella* agar (second generation) plates. Before ENR treatment, *E. coli* E2 and SE211 strains had colonized the GI tracts of chickens. *E. coli* E2 reached levels of 10^5^∼10^7^ CFU per g of feces (Figure 3). SE211 reached levels of 10^3^∼10^5^ CFU per mL of feces. The chickens infected with the SE211 strain showed a somnolent state and cold sensitivity and also passed out green and white loose feces. The *E. coli* E2 strain was still detected in the ENR treatment period and within 3 weeks after termination of ENR treatment. In the NTC group, however, *E. coli* E2 was not detected on day 21 after termination of ENR treatment (Figure 3). This result suggests that the resistant *E. coli* E2 strain could persist in the intestinal tract of chickens under the selection pressure of ENR. In this study, although *E. coli* E2 was isolated from the GI tracts of chickens (Li J. et al., 2019), it was easily excreted from the chicken intestine without the selective pressure of antibiotics. Then, the copy number of the *qnrS* gene was determined to evaluate the prevalence of the resistance gene in the chicken intestinal tract. The standard curve is C_T_ = –3.237 lgC_0_ + 37.172, *R*^2^ = 0.998, Efficiency = 103.693%. This standard curve is credible. In the NTC group, the copy number of *qnrS* gene was higher than the initial detection level on days 1 (1 day after ENR treatment), 3, 7, S.4 (4 days after termination of ENR treatment), S.6, S.14, and lower than the initial detection level on days 5, S.2, S.8, S.21, indicating that the *qnrS* gene could persist in feces (Figure 4). In the prophylactic dose group, the copy number of the *qnrS* gene in the intestinal tracts of chickens showed a downward trend within 3 days of ENR treatment. From days 3 to S.4, the copy number of *qnrS* gene tended to a stable state. From days S.4 to S.21, the copy number of *qnrS* gene increased slightly, but it was still lower than the level before ENR treatment. In the clinically recommended dose group, the copy number of *qnrS* gene was lower than that time point before ENR treatment except on days 7 and S.2. In the high-dose group, the copy number of *qnrS* gene was higher than that time point before ENR treatment from the duration of ENR treatment to day S.4. Within 5 days of ENR treatment, the prophylactic and therapeutic doses of ENR reduced the copy number of *qnrS* resistance gene in chicken intestinal microflora. The high dose of ENR increased the copy number of the *qnrS* gene. Similarly, in the clinically recommended dose group, the copy number of the *qnrS* gene had an increasing trend during 5–7 days of ENR treatment. Therefore, high-dose (50 mg/kg b.w.) and long-term clinically recommended dose (>5 days) ENR treatment increases the risk of *qnrS* gene transmission. All of the *Salmonella* and putative SE211-*qnrS* strains were screened from selective agar plates supplemented with ENR (0.25 mg/L). In ENR treatment duration, no SE211strain was isolated from the high-dosage group. However, the SE211 and putative SE211-*qnrS* strains were isolated from the other groups. The number of putative SE211-*qnrS* strains were changeful. Prior to ENR treatment, no SE211-*qnrS* was detected on ENR-supplemented plates. After 1 day of ENR treatment, one SE211-*qnrS* strain was acquired in the prophylactic dose group. After 2 days of ENR treatment, three SE211-*qnrS* strains were obtained in the clinically recommended dose group. One SE211-*qnrS* strain was isolated from the non-treated control group. After termination of ENR treatment, four SE211-*qnrS* strains were obtained from the clinically recommended dose group (Table 2).

**TABLE 2:** The isolates of putative SE211-*qnrS* obtained.

Sampling day	Group [n/(Total)]			
	NTC	3.03 mg/kg b. w.	10 mg/kg b. w.	50 mg/kg b. w.
1	0 (15)	1 (15)	0 (12)	–
2	0 (12)	0 (9)	3 (15)	–
3	1 (9)	0 (15)	0 (12)	–
S.2	0 (9)	0 (15)	3 (9)	–
S.21	–	–	1 (3)	–

*S, presents discontinue medication; n, number of SE211-qnrS strains; Total, total number of Salmonella isolates; –, no Salmonella isolates; NTC, presents non-treated control group.*

### Sensitivity of SE211-*qnrS* to Four Antibiotics

The resistance phenotypes of SE211-*qnrS* strains were similar to that of the donor strain *E. coli* E2, which exhibited the MDR phenotype as *E. coli* E2 (Table 3). 9 SE211-*qnrS* strains also obtained the *bla*_TEM–35_, *dfrA*-14, *floR*, and *tet*(A) genes located on the IncY plasmid.

**TABLE 3:** MIC of four antibiotics to E2, SE211, and SE211-*qnrS.*

The source of strains	The name of the strains	MIC (mg/L)			
		ENR	AMP	TET	CHL
Donor	E2	128	>256	128	128
Recipient	SE211	0.25	0.5	1	2
NTC	3-9-2	64	>256	128	128
Group 3.03	1–14	64	>256	64	128
	2-29-1	64	> 256	128	128
	2-29-2	128	> 256	128	128
	2-29-3	64	> 256	128	128
Group 10	S.2-23-1	128	> 256	128	128
	S.2-23-2	64	> 256	128	256
	S.2-23-3	128	> 256	128	256
	S.21–24	64	> 256	128	128

*ENR, enrofloxacin; AMP, ampicillin; TET, tetracycline; CHL, chloramphenicol; NTC, non-treated control group; Group 3.03, prophylactic dose (3.03 mg/kg b. w.) of ENR; Group 10, clinical recommended dose of ENR (10 mg/kg b. w.).*

### Instability of Plasmid in *Salmonella*

The SE211-*qnrS* single colony was not recovered from the glycerol bacteria of 26 SE211-*qnrS* (17 *in vitro* and 9 *in vivo*). Although we identified two SE211-*qnrS* strains with positive *repA* and *dfrA*-14 genes in the screening process, they were still sensitive to trimethoprim/sulfamethoxazole when they were tested for the sensitivity to trimethoprim/sulfamethoxazole by glycerol bacteria resuscitation again. It suggested that the two strains also lost the IncY plasmid.

## Discussion

### Subinhibitory Concentration of Enrofloxacin Promotes the Transfer of *qnrS* Gene

PMQR genes can be transmitted among bacteria through horizontal gene transfer (HGT). In this study, transfer of *qnrS* gene from *E. coli* E2 to SE211 was observed both *in vitro* and *in vivo*. Under the laboratory condition, the transfer frequency slightly increased with the increasing concentration of ENR, which might be correlated with the upregulation of conjugation-associated gene expression (Shun-Mei et al., 2018). In this experimental model of intestinal colonization in chickens with MDR *E. coli* E2 and sensitive SE211strains, more SE211*-qnrS* strains were isolated from the ENR-treated group than that from the non-treated control group. This suggests that the resistance gene *qnrS* can be easily transferred to *Salmonella* strains *in vivo* under selective pressure of antibiotics. However, one SE211*-qnrS* strain was also obtained from the NTC group. This indicates that transfer of *qnrS* gene in the intestinal tract of chicken might occur among different bacteria under natural conditions (Gosling et al., 2012), and this kind of horizontal transfer behavior was independent with antibiotic pressure (Le Devendec et al., 2011).

### Enrofloxacin Accelerates the Emergence of Resistant Bacteria

The abuse of antibiotics in poultry and the residues of antibiotics in animals and the environment play a considerable role in the development of resistance among zoonotic food-borne microorganisms (Racewicz et al., 2020). The half-life of ENR is 59.1 days/115.0 days, 88.9 days, and 190.8 days in the dark/light, aerobic, and anaerobic conditions, respectively (Slana and Sollner-Dolenc, 2016). Thus, ENR can exist in chicken feces for a long time. The use of FQs can lead to the increase of resistant strains (Marshall and Levy, 2011). The horizontal transfer of resistance plasmids was the main reason for increasing the resistance level. The treatment of ENR promotes the transfer of the *qnrS* gene to the SE211strain in the intestinal environment of chickens. The isolates of SE211-*qnrS* from the ENR treatment groups were more than that from the NTC group, which confirms the hypothesis that ENR promotes *qnrS* gene transfer. In addition, antibiotics can promote the transfer of resistance genes in chickens (Chen et al., 2016). Similarly, horizontal transfer of plasmid-encoded resistance determinants is reported in the animal intestinal tract under both the presence and absence of antibiotic selective pressure, and the usage of florfenicol and ENR can facilitate the transmission of resistance gene *oqxAB* (Chen et al., 2016). Trimethoprim significantly increased both the HGT and vertical gene transfer frequencies (Li B. et al., 2019). Furthermore, the persistence of antibiotics may lead to the maintenance of resistant bacteria and resistance genes. The colonization results of *E. coli* E2 indicates that ENR can maintain the persistence of *E. coli* E2 carrying *qnrS* gene. It also indicates that treatment with ENR can increase the number of resistant *E. coli* in chicken gut (Roth et al., 2017). Therefore, it is necessary to continuously strengthen the monitoring of resistance genes in clinical strains and take effective measures to eliminate resistance genes and plasmids so as to prevent the spread of resistance genes from aggravating clinical drug resistance.

### *QnrS* Cotransferred With Other Resistance Genes

The other resistance genes coexisting with the *qnrS* gene on the IncY plasmid were generally β-lactam-resistance genes. In the reported IncY plasmid carrying *qnrS* gene, it usually came from *E. coli* in the food chain (Roschanski et al., 2017) and healthy people (Mshana et al., 2016). Similar to this study, the IncY plasmid carried MDR genes to mediate MDR. IncY plasmid as a repository of MDR genes, *E. coli* can pose a threat to human health through the food chain. Here, we found that the *qnrS* gene was cotransferred with the other resistance genes (*bla*_TEM–35_, *tetA*, *floR*, and *dfrA*-14) on the IncY plasmid. In *E. coli*, cotransfer of the *qnrS* gene with β-lactam-resistance genes is reported (Wu et al., 2008; Jiang et al., 2012, 2014). pTET-GZEC065, pTetA_020022, pE2 plasmids had high similarities, carrying different resistance genes, which might be Tn3-family members that confer resistance to antibiotics (Nicolas et al., 2015). Recently, there are many identified Tn3 family members with different combinations of antibiotic-resistance determinants (Stokes and Gillings, 2011; Nordmann et al., 2012). Antimicrobial susceptibility tests show that the resistance spectrum of the SE211*-qnrS* strain was similar to that of donor strain *E. coli* E2 (Table 3). The resistance phenotypes were consistent with the resistance genotypes. In addition, we also obtained a drug-resistant *Salmonella* strain (MIC_ENR_ = 4 mg/L) from the intestinal tract of chicken without the IncY plasmid and *qnrS* gene. We speculate that the resistance mechanism may be attributed to mutations in the *parE* gene. The emergence and increase of MDR bacteria pose a great threat to public health.

### IncY Plasmid Instability in Conjugant

The level of conjugated plasmid-mediated drug resistance in *Salmonella* was found to be slightly higher than that in *E. coli* (Chen et al., 2018). Our research also confirms this finding. In this study, MIC_ENR_ of SE211-*qnrS* strains were 64 or 128 mg/L, whereas MIC_ENR_ of EC600-*qnrS* strain was 8 mg/L (Li J. et al., 2019). The plasmids generally impose a fitness cost on their hosts (Carroll and Wong, 2018). The replication of plasmids in the host bacteria caused metabolic load, and the expression of plasmid-encoded genes was one of the important reasons resulting in the metabolic load of plasmids in the host bacteria (Silva et al., 2012). The expression of plasmid-encoded resistance genes led to the stress response of the host bacteria, resulting in the loss of plasmids (Rozkov et al., 2004). In this study, IncY plasmid carried *qnrS*1, *tet*(A), *floR*, *dfrA-*14, and *bla*_TEM–135_ resistance genes and mediated the resistance of ENR, TET, CHL, TMP, and AMP. Compared with the reported IncY plasmid carrying *qnrS*, the pE2 resistance spectrum was larger. The expression of MDR genes in IncY plasmid undoubtedly increased the metabolic burden of the host bacteria. In addition, the plasmid also had several insertion elements IS421, IS1, ISkpn19, IS26, IS5, and IS91; integron functional elements IntI1; and transposon Tn3, the expression of IS elements and transposons caused the instability of plasmid DNA structure (Haddadin and Harcum, 2005). The copy number of plasmids was also one of the factors determining the metabolic load of plasmids, and the selective pressure of antibiotics was an important condition for maintaining the stability of resistance plasmids. Plasmids with high copy numbers, especially those containing the β-lactam-resistance gene, were unstable and lost quickly in *Salmonella* without antibiotic selection pressure both *in vivo* and *in vitro* (Zhang et al., 2005). In this study, we did not obtain SE211-*qnrS* colonies on plates supplemented with ENR. Although the *repA* and *dfrA*-14 genes of two SE211-*qnrS* strains were positive in the screening process, they were still sensitive to trimethoprim/sulfamethoxazole following glycerol bacteria resuscitation (data not shown). We speculate that antibiotic-free glycerol preserved SE211-*qnrS* strains might easily lose the IncY plasmid. In this study, loss of the IncY plasmid in SE211 might be caused by the absence of antibiotic selective pressure. Thus, antibiotics should be appropriately added to the glycerol bacteria of *Salmonella* transconjugants.

## Conclusion

The *QnrS* gene located on IncY plasmid can transfer from *E. coli* E2 to *Salmonella* SE211 both under laboratory conditions and in the chicken intestinal environment. Sub-MIC of ENR and the clinically recommended dose of ENR can promote conjugation transfer of the *qnrS* gene. The selective pressure exerted by ENR on the intestinal environment of chickens contributed to the persistence of resistance *E. coli* and *qnrS* genes, thus increasing the risk of resistance gene transmission as well as the resistance gene reservoir.

## Data Availability Statement

The authors acknowledge that the data presented in this study must be deposited and made publicly available in an acceptable repository, prior to publication. Frontiers cannot accept a manuscript that does not adhere to our open data policies.

## Ethics Statement

The animal study was reviewed and approved by the study *in vivo* was carried out in accordance with the guidelines established by the China Regulations for the Administration of Affairs Concerning Experimental Animals (1988) and Regulations for the Administration of Affairs Concerning Experimental Animals in Hubei province (2005) (Project No. 2017YFC1600100 and Animal Welfare Assurance No. HZAUCH- 2020-0005). Written informed consent was obtained from the owners for the participation of their animals in this study.

## Author Contributions

MD conceived and designed the study, wrote, reviewed, and edited the manuscript. YZ, ZC, LC, TH, KG, and FZ performed the experiments. YZ and ZC wrote the draft manuscript. All authors participated in the interpretation of the results and read and approved the manuscript.

## Conflict of Interest

The authors declare that the research was conducted in the absence of any commercial or financial relationships that could be construed as a potential conflict of interest.

## Publisher’s Note

All claims expressed in this article are solely those of the authors and do not necessarily represent those of their affiliated organizations, or those of the publisher, the editors and the reviewers. Any product that may be evaluated in this article, or claim that may be made by its manufacturer, is not guaranteed or endorsed by the publisher.

## Footnotes

1
http://www.who.int/news-room/fact-sheets/detail/e-coli

2
https://wwwnc.cdc.gov/travel/yellowbook/2020/travel-related-infectious-diseases/salmonellosis-nontyphoidal

3
http://www.genomicepidemiology.org/index.html

4
https://sourceforge.net/projects/brig/
